# Calling for a more coherent policy response to driving harm

**DOI:** 10.1093/heapro/daag026

**Published:** 2026-03-05

**Authors:** Kate Gray, Grant Ennis, Greg Fell

**Affiliations:** Yorkshire and the Humber School of Public Health, 7-8 Wellington Place, Leeds, LS1 4AP, United Kingdom; Faculty of Science, Monash University, 13 Rainforest Walk, Clayton Campus, Victoria, 3800, Australia; Sheffield City Council, Public Health, Sheffield Town Hall, Sheffield S1 2HH United Kingdom

## Abstract

High rates of population-level driving are associated with multiple adverse health outcomes arising from road crashes, air and noise pollution, physical inactivity, global warming, and social and economic isolation. However, population-level driving is not widely treated as a public health issue, and efforts to reduce driving harms focus on lowering the prevalence of specific high-risk behaviours and exposures (e.g. speeding or drink-driving), not on reducing overall levels of population-driving itself. Silence on driving harms pervades because driving and cars have become deeply embedded in our surroundings, in our policies, and in our social fabric. Urban planning approaches prioritize mobility over access, and the ever-increasing road capacity, combined with underinvestment in walking and sustainable transport, has made society car dependent. Thanks to the influence of the road lobby, the harms of driving are underestimated and overlooked, while measures that make walking, cycling, and public transport the easiest option are viewed with suspicion. Policy responses to reducing population-level driving, as measured by vehicle kilometres travelled per capita and relative mode share, can create positive change. Efforts should focus on (i) improving population-level accessibility—by ensuring shops, schools, and public facilities are close to where people live and work; (ii) making walking and cycling possible and desirable; (iii) realigning incentives for transport so that walking, cycling, and public transport are cheaper and easier than driving; and (iv) communicating effectively and deliberately about transport, health, and happiness.

Contribution to Health Promotion

- Population-level driving, as expressed by the vehicle kilometres travelled (VKT) per capita and relative mode share, is a major public health issue. It contributes to ill-health through air pollution, road crashes, noise pollution, and greenhouse gas emissions and by causing social and economic exclusion.
- However, driving is widely overlooked as a public health issue, partly due to the efforts of the road lobby, the erosion of infrastructure to enable active and sustainable transport, and the politicization of transport policy.
- This paper suggests four policy approaches for addressing driving harm: (i) increase access, (ii) improve walking infrastructure, (iii) reduce driving subsidies, and (iv) smarter framing.

## A gap in our evolving understanding of harm

Our understanding of what constitutes a public health harm has evolved considerably over the last 100 years. However, despite a shift of focus from the control of harmful pathogens to the control of harmful products, environments, and commercial practices, there remain blind spots. There are harms we fail to recognize and address. Chief amongst these is the dose–response relationship between population-level driving and health harm.

## Taking a population-level view of driving and driving harms

While the dominant narrative around driving focuses on individual behaviours and health risks (e.g. the risk of personal injury associated with drink-driving), a more accurate view focuses on ‘population’ driving behaviours and harms. For example, population-level comparison of the number of vehicle kilometres travelled (VKT) per person exposes stark differences between and within countries and reveals a strong relationship between VKT and road deaths per 100 000 (Litman 2017).

High rates of population-level car use harm health through multiple mechanisms. These include road crashes, air and noise pollution, physical inactivity, global warming, environmental degradation, and social and economic isolation (Miner *et al*. 2024).

With road injuries representing the leading global cause of death for 5–19-year-olds (WHO 2023), road crashes are the driving harm that policymakers are most prepared to address. Major gains have been made by reducing speeds in some settings, but with per capita road crash rates increasing with per capita vehicle travel, even more could potentially be achieved through reducing population-level driving. Road danger reduction requires a paradigm shift. Current approaches assume car travel is broadly safe and desirable, with a corresponding focus on strategies to reduce high-risk activities such as speeding and drink-driving. More effective would be an approach that recognizes that all vehicle travel has population health risks and focuses on shifting the curve by reducing unwanted or avoidable motor vehicle journeys while increasing walking and other modes (Litman 2025).

While road crashes attract policy action, there are other less obvious direct driving harms, which are often overlooked. Road transport accounts for 35% of nitrogen oxide emitted in the UK, and vehicle emissions have been linked to heart disease, lung cancer, cognitive impairment, and respiratory conditions (DHSC 2022). Exposure to air pollution from driving is not equally distributed across society with the greatest burden borne by populations who drive the least.

Population-level car dependency also contributes to high levels of physical inactivity and associated health risks. Unsurprisingly, children who are driven to school are less likely to meet the daily activity recommendations (Faulkner *et al*. 2009). Noise pollution from driving is linked to an increased risk of heart disease, stroke, and obesity, with night-time noise a particular risk for cardiovascular disease (WHO 2011).

High rates of population-level driving also bring a set of indirect harms. Global warming is widely recognized as the biggest threat of the 21st century, and globally, transport is responsible for one-quarter of greenhouse gas emissions (United Nations 2021). In the UK, 69% of CO_2_ emitted from all modes of transport came from private vehicles (DfT 2023). Driving is expensive, and low-income households spend a significant portion of their income (as much as 25% in the UK) on it (Massy-Chase 2025). Poorer households also have lower rates of car ownership and are more disadvantaged in settings where social, employment, and education opportunities, as well as basic services, can only be accessed by car.

These issues are acknowledged by some within the public health community, but outside the work of dedicated experts, there is a failure to connect the dots, leading to an incoherent policy response.

## Why are driving harms overlooked?

Silence on driving harms pervades for several interrelated and reinforcing reasons.

1. **The influence of the road lobby is considerable.** The road lobby has fought for the dismantling of rail, against public transport, in favour of unnecessary road building, and has achieved laws that subsidize driving and effectively prohibit active travel by creating legal hurdles to establishing offices, parks, government and commercial spaces walking distance from residential areas and requiring homes be far from one another through such mechanisms as set-back laws and single faming zoning (Miller *et al*. 2025). Just as the tobacco industry invested heavily in normalizing smoking, so too the road lobby has fought to embed a strong social bias towards cars, known as ‘motonormativity’ (Walker *et al*. 2023) via an annual global advertising spend of at least $42.4 billion. Tactics deployed by the road lobby (including focusing on individualized behavioural problems and the promotion of ‘silver bullets’ such as electric cars) will be familiar to those working on other commercial determinants of health (Ennis 2023).
2. **In many cases, people have been given no alternative to driving.** Only half the world’s urban population has convenient access to public transportation (United Nations 2023), and walkable neighbourhoods with close access to daily essentials are increasingly scarce. Motor-industry-friendly urban planning, such as laws that incentivize low densities, lane width minimums rather than maximums, or parking minimums, increasingly makes society dependent on cars and has contributed to forced car ownership (Mattioli 2017).
3. **It is political.** In some contexts, such as the UK, driving has become politicized. Resource allocations for more road space for driving are presented as technical, apolitical, and necessary for economic growth. Conversely, interventions to encourage active travel and disincentivize car use are portrayed as ‘woke’, and anti-growth (Bor 2025), and are ripe for political challenge, making them too-hot-to-handle.

## What policy approaches can be taken?

If we continue to build neighbourhoods dependent on cars, high mileage (or kilometres) per capita and driving harm will continue to grow. However, change is possible as demonstrated in settings such as Paris. Achieving change requires the following:

1. **Creating high-access neighbourhoods.** Rather than prioritizing mobility—which means optimizing speeds—we can switch focus to prioritize access. Rather than building Dubai-style spaghetti road networks in the hearts of our communities, we can build denser multi-use neighbourhoods and provide tax and regulatory incentives to businesses, schools, and public services to open near to where people live—so that driving is less necessary and access is improved.
2. **Making walking and cycling possible and desirable.** Pedestrian infrastructure in the form of footpath networks, narrowed car lanes, and raised crosswalks serve to make it safer to walk and cycle. Implemented alongside interventions that discourage driving for short journeys, these approaches have been shown to shift mode share from driving to active travel (Xiao *et al*. 2022).
3. **Realigning incentives for transport to make driving less easy.** Governments worldwide increasingly encourage driving via a myriad of incentives that make it significantly cheaper than active or public transport, such as subsidized parking, energy, vehicles, and roads (Shill 2020). Systematically eliminating these perverse incentives will make it easier to access the important things in life by foot or by public transport, rather than by car (Anciaes *et al*. 2025).
4. **Be aware of and challenge car-centred (or car industry) framing.** The way we explain and think about problems defines the solutions we use to address them. Currently, much of the conversation around transport further entrenches motonormativity. Rather than focusing on negatives such as ‘low traffic neighbourhoods’ or ‘closed streets’, we can test more positive, values-based framings, which emphasize community, access, health, and happiness. In doing so, we adjust social norms and depoliticize effective policy interventions.

## Harm is harm

Driving harm is a major public health issue that has been overlooked and is addressed in a piecemeal way by policymakers. There is a dose–response relationship between the level of societal driving as a share of transport mode and total vehicle distance travelled and the harms resulting from driving. Similar to the consensus that there being ‘no safe lower limit’ to alcohol consumption, there is no ‘harmless’ level of driving. To reduce driving harm, driving itself needs to be reduced both by creating a built environment conducive to other modes and by reducing the incentives for driving. Harm is harm, and driving harm needs to be considered alongside the other big commercial killers of our age.

## Data Availability

No new data were generated or analysed in support of this work.
